# Eumelanin from the Black Soldier Fly as Sustainable Biomaterial: Characterisation and Functional Benefits in Tissue-Engineered Composite Scaffolds

**DOI:** 10.3390/biomedicines10112945

**Published:** 2022-11-16

**Authors:** Ugo D’Amora, Alessandra Soriente, Alfredo Ronca, Stefania Scialla, Martina Perrella, Paola Manini, Jun Wei Phua, Christoph Ottenheim, Rocco Di Girolamo, Alessandro Pezzella, Maria Grazia Raucci, Luigi Ambrosio

**Affiliations:** 1 Institute of Polymers, Composites and Biomaterials, National Research Council, 80135 Naples, Italy; 2 Department of Chemical Sciences, University of Naples Federico II, 80126 Naples, Italy; 3 Bioelectronics Task Force, University of Naples Federico II, 80126 Naples, Italy; 4 Insectta, 60 Jalan Penjara, Singapore 149375, Singapore; 5 Department of Physics “E. Pancini”, University of Naples Federico II, 80126 Naples, Italy

**Keywords:** eumelanins, hyaluronic acid, methacrylated hyaluronic acid, 3D printing, scaffolds, bone tissue engineering

## Abstract

An optimized extraction protocol for eumelanins from black soldier flies (BSF-Eumel) allows an in-depth study of natural eumelanin pigments, which are a valuable tool for the design and fabrication of sustainable scaffolds. Here, water-soluble BSF-Eumel sub-micrometer colloidal particles were used as bioactive signals for developing a composite biomaterial ink for scaffold preparation. For this purpose, BSF-Eumel was characterized both chemically and morphologically; moreover, biological studies were carried out to investigate the dose-dependent cell viability and its influence on human mesenchymal stem cells (hMSCs), with the aim of validating suitable protocols and to find an optimal working concentration for eumelanin-based scaffold preparation. As proof of concept, 3D printed scaffolds based on methacrylated hyaluronic acid (MEHA) and BSF-Eumel were successfully produced. The scaffolds with and without BSF-Eumel were characterized in terms of their physico-chemical, mechanical and biological behaviours. The results showed that MEHA/BSF-Eumel scaffolds had similar storage modulus values to MEHA scaffolds. In terms of swelling ratio and stability, these scaffolds were able to retain their structure without significant changes over 21 days. Biological investigations demonstrated the ability of the bioactivated scaffolds to support the adhesion, proliferation and osteogenic differentiation of human mesenchymal stem cells.

## 1. Introduction

**Figure 1:**
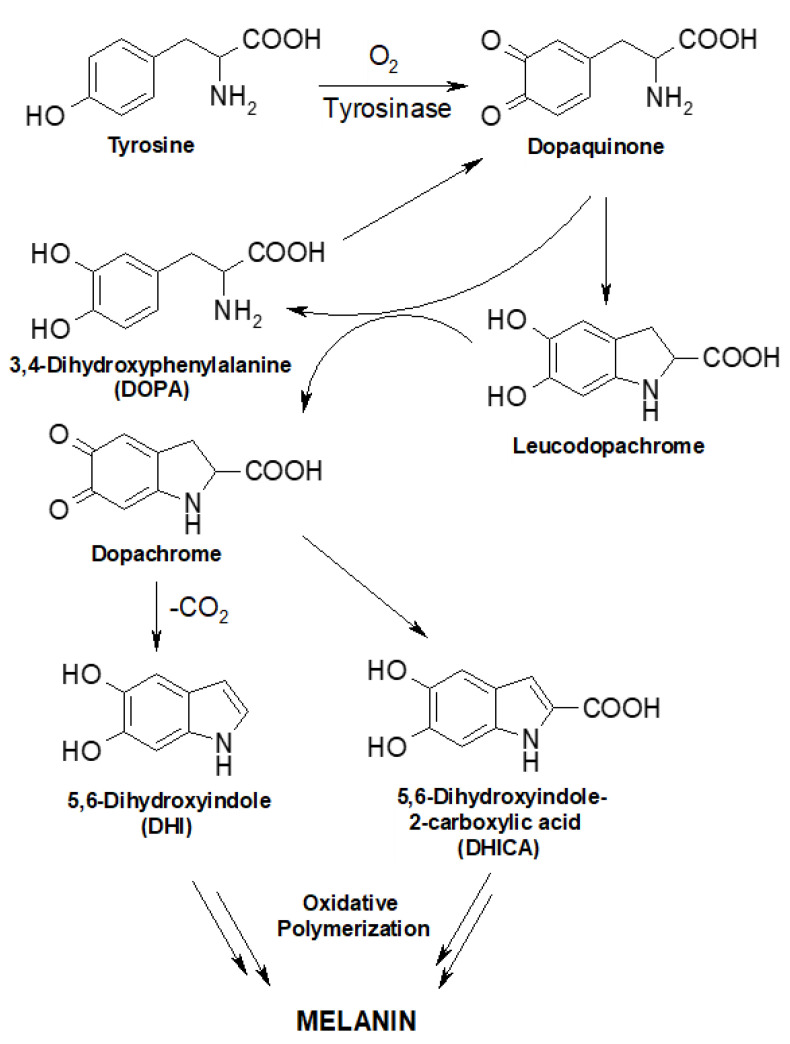
Biosynthetic pathway of eumelanins.

Eumelanin biopolymers are emerging as versatile tools for the design and fabrication of smart materials in a number of different research fields, from organic electronics to biomedicine and thus in bioelectronics [1,2,3,4]. Eumelanins are a class of natural nitrogen containing pigments responsible for the brown to black coloration throughout the animal kingdom; they are commonly found in mammals as well as the exoskeleton of many insects [5,6]. Eumelanins are formed by the oxidative polymerization of 5,6-dihydroxyindole derivatives, DHI (5,6-dihydroxyindole) and DHICA (5,6-dihydroxyindole-2 carboxylic acid, arising through the oxidative metabolism of the L-tyrosine amino acid (Figure 1) [7].

Eumelanins exhibit a peculiar set of physico-chemical [8] such as: (1) a broadband and featureless UV-visible absorption profile responsible for their dark colour; (2) a persistent free radical character denoted by an intense signal in the electron paramagnetic resonance (EPR) spectrum; (3) a water-dependent hybrid ionic−electronic conductor behaviour; (4) having multiple redox states available. Overall, these properties have stimulated the application of eumelanins in different sectors including organic electronics, with the design of the eumelanin-based organic thin film transistor (O-TFT) [9], organic photovoltaic (OPV) devices [10], organic light-emitting diodes (OLEDs) [11] and supercapacitors [12]; regenerative biomedicine, with the design of eumelanin-based bio-interfaces promoting the adhesion, proliferation and differentiation of several cell lines and stem cells [9]; and remediation processes, with the design of eumelanin-based devices able to sequester metal ions, drugs and pollutants [13].

To date, the majority of the studies addressing the properties and application of eumelanins as innovative materials have been carried out on synthetic polymers obtained by the oxidative polymerization of DHI and/or DHICA under biomimetic conditions [2,3]. Only a few examples are reported in the literature dealing with the natural pigments as extracted from natural sources, because of the many difficulties associated with the extraction and purification protocols [7,14,15]. Beyond cephalopod inks, insect exoskeletons are another large and important source of eumelanins, and a recent patent by Insectta described a method to obtain valuable amounts of pure eumelanins from black soldier fly (BSF-Eumel) cuticles [16]. In recent years, the farming of BSF (*Hermetia illucens*) has gained attention as a means to obtain proteins and fats for the animal feed industry while valorising both homogeneous and heterogeneous food waste that might otherwise be destined for the incinerator or landfill. Massive farms now exist worldwide, processing hundreds of tons of homogeneous waste streams [17,18]. The life cycle of the BSF consists of five life stages: egg, larva, pre-pupa, pupa and adult. During the pre-pupal stage, the BSF actively produces eumelanin, which may then be found in the fats and the exoskeleton of the insect. This results in the black colouration of the pre-pupal, pupal and adult stages, and is hypothesized to exist as a means to protect against ultraviolet (UV)radiation and as a form of camouflage [19]. During the metamorphosis of the BSF from pupal to adult stage, the highly mineralised pupal exuviae is left behind and is available in large quantities. The development of the BSF farming industry thus opens up exciting possibilities for the large-scale production of eumelanin.

In the regenerative medicine field, eumelanin has mainly been employed for wound healing applications [20]. It has also shown interesting properties in stimulating nerve and bone regeneration [14,21]. For example, Yoo et al. showed the positive influence of melanin on bone, by the activation of Bone Morphogenic Protein (BMP) or runt-related transcription factor 2 (RUNX2) signaling pathways and the regulation of osteogenic genes, such as alkaline phosphatase (ALP), type I collagen and osteocalcin [14]. Inspired by this application, a hyaluronic acid sodium salt with high molecular weight (HM_w_HAs)-based matrix was selected for the 3D printing of a bone tissue-engineered scaffold in which BSF-Eumel was supposed to exert a bioactive stimulus. HA [α-1,4-D-glucuronic acid-β-1,3-N-acetyl-D-glucosamine]_n_ is a naturally occurring hydrophilic glycosaminoglycan whose chemical structure may allow easy chemical modification of the hydroxyl groups by esterification [22,23]. Indeed, modification with methacrylate groups can allow the crosslinking by UV light, in the presence of a photoinitiator. Methacrylated HA (MEHA) hydrogels have shown increased mechanical properties and improved resistance to degradation compared to non-modified HA hydrogels while maintaining good biocompatibility [24,25,26,27]. In particular, methacrylation allows conferring a shear thinning behaviour, thus the ability to be 3D printed, maintaining scaffold shape before and after the printing process [24]. For example, Poldervaart et al. successfully demonstrated that cell-embedded HMwMEHA hydrogels were able, per se, to induce intrinsic differentiation. For this reason, the authors employed this material in 3D bioprinted scaffolds combining their high levels of cell survival and intrinsic osteogenic differentiation in a 3D bioprintable hydrogel, which resulted in promising bone tissue engineering [28,29].

Starting from this background, and in view of expanding the scope of eumelanin exploitation for advanced biomaterial design and development, herein is reported for the first time an almost complete characterization of BSF-Eumel and a preliminary study addressing the use of BSF-Eumel as an additive in a biocompatible ink for 3D printing fabrication.

## 2. Materials and Methods

### 2.1. Eumelanin Extraction from Black Soldier Fly (BSF) Cuticles

BSF-Eumel powder was isolated from the black soldier fly (*Hermetia illucens*) according to a patented protocol released by Insectta [16]. Black soldier fly (*Hermetia illucens*) pupal exuviae were purchased from Hermetia Bio Science (Jakarta, Indonesia) and minced into approximately 0.5 mm pieces using a blender (Robot Coupe Blixer 4, France). The pupal exuviae were then demineralised with 10% (*w*/*w*) lactic acid at room temperature for 3 h. In order to reduce protein contamination in the eumelanin fraction, deproteination of the pupal exuviae was performed with 1M sodium hydroxide for 3 h at 50 °C. Subsequently, eumelanin was liberated by heating the pupal exuviae with 3M NaOH, for 2 h at 90 °C. Thorough washing of the pupal exuviae was performed in between steps. The eumelanin-containing supernatant was filtered through a 500 mesh nylon cloth, and the eumelanin was precipitated with the addition of 37% (*v*/*v*) hydrochloric acid. The eumelanin was then pelleted by centrifugation, washed and subjected to a series of proprietary steps, which concluded with lyophilization to obtain salt-free, water-soluble sub-micrometer colloidal particles (BSF-Eumel).

### 2.2. Physico-Chemical and Biological Characterization of BSF-Eumel

#### 2.2.1. Physico-Chemical Characterization

The morphology of BSF-Eumel was investigated by transmission electron microscopy (TEM) using an FEI Tecnai G2 S-twin apparatus operating at 200 kV (LaB6 source) equipped with 4K Eagle camera. BSF-Eumel formulations were prepared by dispersing the obtained powders in aqueous solution and then placing a drop of suspension on one side of the carbon-coated copper grid (200 mesh). The elemental composition (C, H, N % w) was estimated by using a PerkinElmer 2400 CHNSO elemental analyser.

Ultraviolet–visible (UV-vis) spectra were recorded using a PerkinElmer Lambda 900 spectrophotometer.

The electron paramagnetic resonance (EPR) spectra were measured using an X-band (9 GHz) spectrometer (Bruker Elexys E-500). 

Dynamic Light Scattering (DLS) was employed to measure the surface charge (ξ-potential) of BSF-Eumel by using a Zetasizer Nano ZS instrument (Malvern instruments, Malvern, UK). The stability of BSF-Eumel was determined by measuring the variation in the surface charge in different buffers: distilled water (*di*H_2_O), 1X phosphate-buffered saline (PBS, Sigma Aldrich, Milan, Italy) solution, Dulbecco’s Modified Eagle’s Medium-high glucose (DMEM, Sigma Aldrich, Milan, Italy) and DMEM + 10% (*v*/*v*) foetal bovine serum (FBS, Sigma Aldrich, Milan, Italy), pH 7.4, at room temperature. BSF-Eumel was diluted in each buffer 1:100 (*v*/*v*), in order to obtain a signal of 100–500 Kcps, and measured using a carbon electrode cell. The system operates in backscattering mode with a He-Ne laser beam (λ = 532 nm) at 25 °C; the scattering angle was set at 173° with a stabilization time of 60 s, and considering the refractive index of BSF-Eumel (1.650).

#### 2.2.2. Cell Viability and Morphology

Biocompatibility tests were performed on BSF-Eumel at different concentrations by using murine fibroblasts cell line as an in vitro model (L929; ECACC 85011425, mouse C3H/An). Before starting cell viability evaluation, the BSF-Eumel solution was sterilized by filtration method using a 0.22 µm membrane filter. BSF-Eumel biocompatibility was determined by testing the cell viability in a dose-dependent manner (2.5, 1.25, 0.625, 0.3125, 0.125 and 0.05 mg/mL serial dilution). To this aim, L929 cells were cultured in plastic culture dishes in DMEM, supplemented with 10% (*v*/*v*) FBS, antibiotic solution (streptomycin 100 µg/mL and penicillin 100 U/mL, Sigma Aldrich, Milan, Italy) and 2 mM L-glutamine (Sigma Aldrich, Milan, Italy). Cells at a density of 1 × 10^4^ cell/mL were incubated with different BSF-Eumel solutions at 37 °C in a humidified atmosphere with 5% CO_2_ and 95% air humidity for 24 h.

Alamar blue reagent (Life Technologies, Milano, Italy) was used to analyse the cell vitality as function of metabolic activity of healthy cells. In particular, the assay is based on the redox reaction of resazurin dye that in its oxidized form is blue in colour and non-fluorescent. The cell growth allows to preserve a reduced microenvironment (non-fluorescent), while the inhibition of growth maintains an oxidized environment (non-fluorescent). The test was performed following the manufactures’ protocol. Briefly, the cells in contact with BSF-Eumel solutions were treated with Alamar blue solution (10% (*v*/*v*) Alamar blue in DMEM without phenol red) for 4 h at 37 °C and 5% CO_2_. Therefore, the absorbance was detected using a spectrophotometer (VICTOR X3, PerkinElmer, Milano, Italy) at wavelengths of 570 and 600 nm. The cell viability was expressed as percentage of Alamar blue reduction.

Morphology of fibroblast cells was analysed by optical and fluorescence microscopy (JuLI™ Stage Real-Time CHR, (Cell History Recorder)) at 24 h after cell seeding. Non-attached cells were removed by washing with 1X PBS, while the attached cells were permeabilized with Triton X-100 for 1 h and treated with a green phalloidin (1:200, Alexa Fluor R-488, Phalloidin Molecular Probes) for 1 h. Cell nuclei were stained with 10 μg/mL of DAPI solution (Sigma Aldrich, Milan, Italy) for 15 min at 37 °C in phenol red-free medium. Afterwards, the cell culture was washed with 1X PBS and observed by fluorescent microscope.

#### 2.2.3. Early Cell Osteogenic Differentiation: Alkaline Phosphatase Expression 

For alkaline phosphatase (ALP) quantification, human mesenchymal stem cells (hMSCs, 1 × 10^4^ cells/well, ATCC, PCS500012, 70042693) from adult bone marrow at 3rd passage were used. Cells were cultured in DMEM medium, containing 10% (*v*/*v*) FBS, 100 U/mL penicillin and 0.1 mg/mL streptomycin, 200 mM L-glutamine, in a humidified atmosphere at 37 °C and 5% CO_2_. Starting from a preliminary study performed with different BSF-Eumel concentrations, the concentration of 0.3125 mg/mL was selected in order to evaluate the differentiation of hMSCs. The assay was carried out at 7 and 14 days. At each time point, cultures were gently washed with 1X PBS, followed by washing with cold 1X assay buffer (BD Biosciences, Milan, Italy). The cultures were next treated with 1X lysis buffer with 0.2% of Triton X-100 to obtain cell lysates. The ALP activity was then evaluated on the cell lysate (50 µL) using an ALP kit (SensoLyte pNPP ALP assay kit, ANASPEC, Milan, Italy). To correct the ALP values for the number of live cells, double-stranded DNA (dsDNA), as a marker for the cell number, was quantified using a PicoGreen_dsDNA kit (Life Technologies, Milan, Italy), following the manufacturer’s protocol.

The biological samples were excited at 480 nm and the fluorescence emission of Picogreen was determined at 520 nm after excitation using a spectrophotometer. The concentration of dsDNA (ng/mL) was obtained by a calibration curve where the fluorescence emission intensity was plotted versus DNA concentration. The experiment was performed in triplicate four times. The ALP activity was obtained by normalizing the amount of ALP (ng) to the total DNA expressed in micrograms (μg).

### 2.3. 3D Printed MEHA and MEHA/BSF-Eumel Scaffolds

#### 2.3.1. Synthesis and Characterisation of Photocrosslinkable MEHA

Hyaluronic acid sodium salt from *Streptococcus equi* (HAs, High Mw = 1.5–1.8 × 10^6^ Da, Sigma Aldrich, Milan, Italy) was modified to graft photoactive moieties by reacting with methacrylic anhydride (ME; Sigma Aldrich, Milan, Italy). HAs was dissolved in fresh deionized (*di*H_2_O) water (Carlo Erba, Cornaredo, Italy) and stirred overnight at room temperature for complete dissolution. The functionalization was performed according to a protocol previously described [25,27]. Briefly, an excess of 30 mol% ME per hydroxyl group (-OH) was added dropwise to the MEHA solution at room temperature, after which the pH was adjusted between 8 and 9 by adding sodium hydroxide (NaOH, Sigma Aldrich, Milan, Italy). MEHA solutions were precipitated into cold anhydrous ethyl alcohol (EtOH, Sigma Aldrich, Milan, Italy) and the supernatant was recovered by filtration and subsequent centrifugation to remove all the unreacted ME. The obtained MEHA polymer was then dialyzed against *di*H_2_O for 5 days and freeze dried.

#### 2.3.2. Attenuated Total Reflection Fourier Transform Infrared (ATR-FTIR) Spectroscopy

ATR-FTIR analysis was performed by ThermoFisher Nicolet (IS10, Waltham, MA, USA) to identify the functional groups attached to HAs. Dried MEHA was scanned from 600 to 2000 cm^−1^ with resolution of 2 cm^−1^. The neat HAs powder was also tested as control under the same conditions.

#### 2.3.3. Three-Dimensional Printing of MEHA and MEHA/BSF-Eumel Scaffolds

Freeze-dried MEHA was dissolved in *di*H_2_O at a concentration of 4% (*w*/*v*) containing 0.1% (*w*/*v*) 2-hydroxy-4′-(2-hydroxyethoxy)-2-methylpropiophenone (Irgacure 2959, Sigma Aldrich, Milan, Italy), the latter of which was used as a biocompatible photoinitiator. Similarly, for MEHA/BSF-Eumel, MEHA (4% (*w*/*v*)) was dissolved in an aqueous solution of BSF-Eumel at the concentration of 0.3125 mg/mL, which also contained Irgacure.

**Figure 2:**
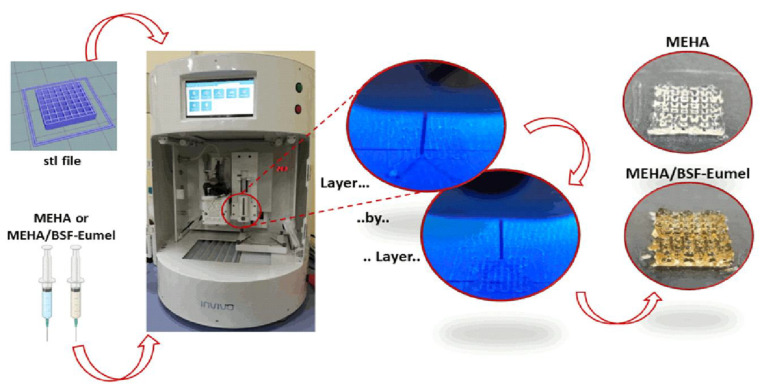
Fabrication steps from biomaterial ink synthesis to the 3D printed MEHA and MEHA/BSF-Eumel scaffolds.

Three-dimensional printing was performed on “Rokit Invivo 4D2” (Rokit Healthcare Inc., Seoul, Korea) using 1.80 firmware. The input printing model was sliced with a grid pattern using NewCreatorK 1.57.70. The neat and composite inks were loaded in a Luer-Lock glass syringe (10 mL) with 15.5 mm inner diameter. The printing speed was set at 6 mm/s. The dispenser temperature was set at 15 °C, while the bed was set at 0 °C. A needle of 0.6 mm, a layer thickness of 0.4 mm and a fill density of 70% were used to build a cubic shape structure of 15 mm × 15 mm × 3 mm for mechanical and 5 mm × 5 mm × 3 mm for biological analyses as shown in Figure 2.

During printing, UV light (λ: 365 nm) was used to crosslink the biomaterial ink enhancing the mechanical properties and avoiding the collapse of the structures. After printing, 3D porous structures were subjected to a post-crosslinking step in a UV cabinet (Analytik Jena UVP crosslinker, λ: 365 nm, P: 10 J/cm^2^) for 10 min to ensure the complete conversion of methacrylate moieties.

#### 2.3.4. Dynamic Mechanical Analysis (DMA) 

TA-Q800 (TA-Instrument, New Castle, DE, USA) was employed to assess the mechanical properties of 3D printed structures. The frequency was varied between 0.5 and 10 Hz, simulating the physiological stride. Furthermore, an amplitude of 100 µm in compression, a preload of 0.001 N and a force track of 125% were adopted. The tests were performed in a closed chamber in a wet state, and at room temperature. 

#### 2.3.5. Swelling Studies and Stability Tests

Freeze-dried MEHA and MEHA/BSF-Eumel scaffolds were weighted (w_0_) and left to swell in physiological conditions up to 21 days (pH = 7.4, T = 37 °C) in 1 mL *di*H_2_O. To assess their ability to retain water, the swollen hydrogels were then taken out at selected time points, the weight was recorded (w_t_) and the samples re-placed in *di*H_2_O. The swelling ratio (Q) was calculated according to Equation (1):

Q = (w_t_ − w_0_)/w_0_ (1)

The stability of the scaffolds was assessed by immersing the samples in physiological conditions (pH 7.4, 37 °C) for up to 21 days. At fixed time points, the scaffolds were removed and their weight was recorded. The remaining scaffold’s weight was calculated using Equation (2):

Remaining weight% = w_t_/w_d1_ × 100% (2)

where w_t_ and w_d1_ indicate the weight of the scaffolds at the selected time intervals and at day 1, respectively.

#### 2.3.6. Release Study of BSF-Eumel from MEHA Scaffolds

MEHA/BSF-Eumel scaffolds were incubated in 1 mL of *di*H_2_O and kept at 37 °C for up to 15 days. For release kinetics, the supernatant was collected at different time points (5 h, 3, 6, 8, and 15 days), and 1 mL of fresh *di*H_2_O was added to each sample to replace the extracted liquid. The supernatant was lyophilized and resuspended in 1 mL *di*H_2_O and the absorbance measured at 490 nm using a UV-Vis spectrophotometer (Victor X3 Multilabel Plate Reader, PerkinElmer-IT). To convert the absorbance value to the amount of melanin, a standard curve was obtained.

#### 2.3.7. Biological Analyses 

Biological investigations on 3D printed scaffolds were performed by using hMSC cell lines. MEHA and MEHA/BSF-Eumel scaffolds were sterilized overnight by soaking in 70% (*v*/*v*) ethanol and by exposure to UV for 2 h, each side. 

#### 2.3.8. Cell Viability and Osteogenic Differentiation

hMSCs (1 × 10^4^ cells/scaffold) were seeded on MEHA and MEHA/BSF-Eumel to evaluate the effect of materials on cell viability and differentiation in basal medium (DMEM supplemented with 10% (*v*/*v*) FBS, antibiotic solution (streptomycin 100 μg/mL and penicillin 100 U/mL) and 2 mM L-glutamine) for 7 and 14 days of cell culture. Cell proliferation and differentiation for MEHA and MEHA/BSF-Eumel scaffolds were analysed as described above by using Alamar blue and ALP/DNA assays, respectively.

### 2.4. Statistics and Data Analysis

For each experiment, at the very least, triplicate specimens were tested, unless otherwise stated. Results are presented as mean ± standard deviation (S.D.) of independent measurements. For the statistical analysis, one-way or two-way ANOVA, followed by Tukey’s post hoc test with multiple comparisons among the different groups, were performed by using GraphPad Prism software (version 7.0). Different levels of significance were considered 95–99.9999% between the different results.

## 3. Results and Discussion

### 3.1. Physico-Chemical and Biological Characterization of BSF-Eumel

Over the last years, eumelanins have been synthetically produced and employed in biomedicine-related areas as a radiation- and photo-protector, as well as a contrast agent [28,29,30,31,32]. Only a few applications have involved tissue engineering and regenerative medicine [14,20,21]. However, in view of a more sustainable and circular economy, the possibility to extract eumelanin pigments from different natural sources has boosted great advances in material science and engineering [7,14,15]. Indeed, exploring naturally-derived biomaterials is attracting outstanding research attention. In this scenario, the company Insectta [16] allowed the synthesis of BSF-Eumel, which was characterized in terms of its physico-chemical, morphological and biological properties.

**Figure 3:**
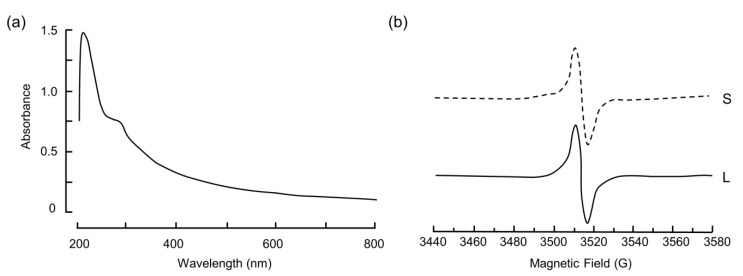
(**a**) UV-vis absorption spectra of BSF-Eumel in PBS, pH 7.4. (**b**) EPR analysis of BSF-Eumel at the solid state (S) and as PBS solution (L).

BSF-Eumel exhibited a good solubility in diH_2_O and aqueous buffers, thus allowing for a more accurate physico-chemical characterization. In particular, the UV-vis spectrum registered in PBS, pH 7.4, exhibited the typical melanin profile characterised by a featureless absorption covering the entire UV and visible regions of the spectrum (Figure 3a) [7]. The EPR spectra registered both on the solid sample and on the corresponding PBS solution confirmed the presence of a slightly asymmetric intense signal (Figure 3b).

Elemental analysis (Table 1) provided clear evidence of an increase in oxygen content in BSF-Eumel with respect to what is typical of the eumelanin precursor 5,6-dihydroxyindole (DHI). This was expected as eumelanins are formed via DHI oxidation; moreover, the extraction protocol can promote the further oxidation of the sample and thus the relative weight increase in oxygen. The high oxidation level of the BSF-Eumel suggested a large availability of oxygen bearing functional groups, which in turn can confer metal chelating attitude to the pigment. The affinity of melanin samples for metal ions is well documented in the literature and is associated with the redox state of the polymer [33].

**Figure 4:**
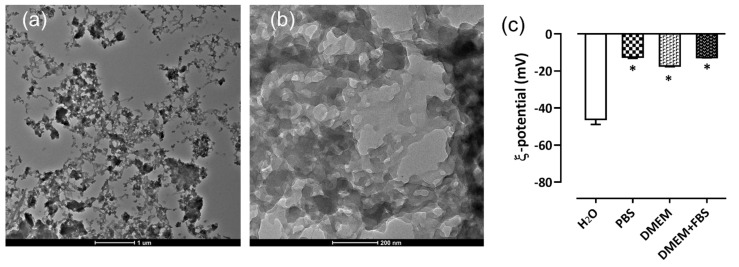
TEM micrographs of BSF-Eumel: (**a**) scale bar: 1 µm, (**b**) scale bar: 200 nm. (**c**) Zeta (ξ) potential of BSF-Eumel as function of different conductivities and ionic strengths of medium (*di*H_2_O, PBS, DMEM and DMEM + 10% (*v*/*v*) FBS) measured by DLS. (* *p* < 0.05).

The morphological analysis of BSF-Eumel carried out by TEM analysis (Figure 4a,b) disclosed a non-homogeneous sub-micrometer colloidal particle distribution, resembling those reported for synthetic melanin polymers [34]. The stability of BSF-Eumel was studied in media with different conductivities and ionic strengths, i.e., *di*H_2_O, PBS. In addition, the BSF-Eumel stability in relation to protein corona formation was also assessed by studying their behaviour in phenol red-free DMEM with and without 10% (*v*/*v*) FBS. The average ξ-potential values, presented in Figure 4c, confirmed the colloidal stability of BSF-Eumel in *di*H_2_O found to be around ×46.1 ± 2.7 mV. A significant reduction in the ξ-potential of BSF-Eumel in the other buffers might be attributed to ion salt absorption (in the presence of PBS and DMEM), as well as a possible protein corona formation around BSF-Eumel, mostly due to the albumin inside the serum.

**Figure 5:**
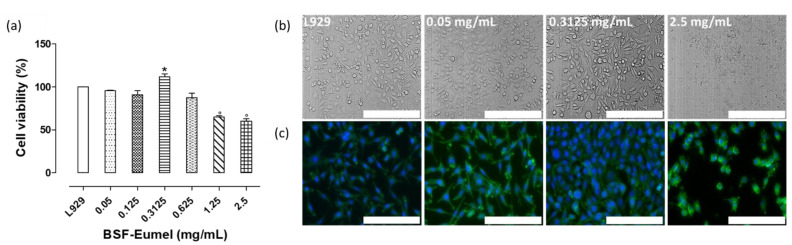
(**a**) Alamar blue cell viability assay (%, normalized to control) performed on L929 cells grown in the presence of BSF-Eumel serial dilution (2.5, 1.25, 0.625, 0.3125, 0.125, 0.05 mg/mL) after 24 h of treatment. Data are presented as mean ± SD. Statistical analysis of variance on mean values is assessed by one-way ANOVA followed by Tukey’s post hoc test with multiple comparisons. (* *p* < 0.05; ° *p* < 0.001). (**b**) Optical micrographs and (**c**) fluorescent images of L929 with and without BSF-Eumel treatment at 24 h of in vitro cell culture (Scale bar: 500 μm).

Finally, an in vitro dose-dependent cell viability study was performed on BSF-Eumel solutions to choose the best working concentration suitable for MEHA/BSF-Eumel biomaterial ink preparation. The cell viability (%) of BSF-Eumel solution at different concentrations, in the range of 0.05–2.5 mg/mL, demonstrated that BSF-Eumel showed no negative effects on cell vitality compared with the control up to the concentration 0.625 mg/mL. However, higher concentrations (1.25–2.5 mg/mL) resulted in a significant reduction of about 30% compared to the control (Figure 5a). The optical and fluorescent micrographs (Figure 5a,b) confirmed the quantitative data. Indeed, good cell vitality and spreading for the selected concentrations (0.05–0.3125 mg/mL) and lowest vitality and round morphology at a higher concentration (2.5 mg/mL) was observed. This study allowed the selection of 0.3125 mg/mL BSF-Eumel as the optimal working concentration.

### 3.2. Three-Dimensional Printed MEHA and MEHA/BSF-Eumel Scaffolds

In regenerative medicine, scaffolds play a pivotal role as support matrixes in guiding tissue formation. They must mimic the native extracellular matrix (ECM), affecting cell behaviour in terms of cell attachment, proliferation and differentiation [32]. A naturally cell-adhesive hydrogel, such as MEHA, has been employed as a 3D printed scaffold matrix [31]. However, the polymer, *per se*, has shown some limitations in perfectly recapitulating the biological environment. Herein, the rationale behind this work was to suggest the use of BSF-Eumel as bioactive signals for developing a composite biomaterial ink for the preparation of a bone tissue-engineered scaffold by 3D printing. 

The use of HMwHAs has been reported for application in bone tissue engineering, but the short degradation time and the poor printability make it not useful for 3D bioprinting [31]. 

**Figure 6:**
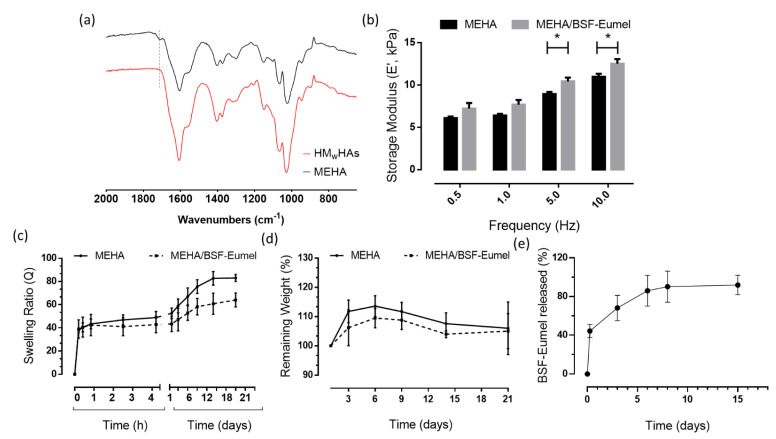
(**a**) ATR-FTIR spectra of HMwHAs and MEHA, highlighting the success of chemical modification. (**b**) Dynamic mechanical analysis performed on MEHA and MEHA/BSF-Eumel scaffolds. * *p* < 0.05. (**c**) Swelling behaviour and (**d**) degradation profile of MEHA and MEHA/BSF-Eumel scaffolds up to 21 days. (**e**) Release profile of BSF-Eumel from 3D printed scaffolds up to 15 days in diH_2_O. Data reported as mean value ± S.D., n = 3.

From this perspective, it has been extensively demonstrated that methacrylation is a possible strategy to improve the physico-chemical and mechanical properties of HAs. Indeed, in this work, methacrylation of HMwHAs allowed the preparation of a biomaterial ink with higher mechanical properties, prolonged degradation time and good printability [31]. To assess the presence of methacrylic moieties on the HA backbone, ATR-FTIR of HAs and MEHA derivatives was performed (Figure 6). In the spectra, two bands were clearly reported: (1) (950 and 1200 cm^−1^), which is related to the C-O stretching vibrations (νC-OH), (2) (1500 to 1700 cm^−1^), which corresponds to the superposition of amide I and II and of various carbonyl and carboxyl νC = O bands. At 1719 cm^−1^, the presence of the peak related to νC = O of the methacrylic group confirms the success of the methacrylation reaction. Once assessed that the reaction was successfully carried out, the proposed MEHA and MEHA/BSF-Eumel biomaterials inks were processed to produce 3D porous scaffolds with a morphologically-controlled structure. The black yellowish colour of the 3D printed MEHA/BSF-Eumel scaffold, in contrast to the white/transparent appearance of MEHA, confirmed the inclusion of BSF-Eumel sub-micrometer colloidal particles into the polymeric structures (Figure 2).

Dynamic mechanical analysis (DMA) was employed to assess the mechanical properties of 3D printed MEHA and MEHA/BSF-Eumel structures. Figure 6b reports the elastic modulus (E’) as a function of the frequency for MEHA and MEHA/BSF-Eumel 3D structures. In particular, MEHA scaffolds showed a value of E’ ranging from 6.0 ± 0.2 kPa (0.5 Hz) to 10.9 ± 0.4 kPa (10 Hz). The E’ value was 6.4 ± 0.2 kPa, at the normal physiological stride frequency of 1 Hz, statistically different from E’ in traumatic conditions (10 Hz), which resulted in 10.9 ± 0.3 kPa, (*p* < 0.001). The presence of BSF-Eumel slightly affected the E’ at a lower frequency. Indeed, E’ was 7.7 ± 0.2 kPa; meanwhile, at higher frequencies (5 and 10 Hz), E’ for MEHA/BSF-Eumel scaffolds was statistically higher than the MEHA one, highlighting storage moduli of 12.5 ± 0.6 kPa and 10.4 ± 0.4 kPa, respectively. However, in the present study, the overall results from mechanical characterization of the 3D printed scaffolds showed values of storage moduli in agreement with those reported in the literature [31]. Specifically, the authors found a value of E’ for bulk hydrogels, which spanned from 1.3 to 10.6 kPa by varying the MEHA concentration in the range of 1 to 3% (*w*/*v*).

The ability to absorb water is an important feature of a tissue-engineered scaffold since swelling may increase pore size, allowing cell infiltration into the scaffolds during in vitro cell culture [35]. In terms of swelling behaviour, MEHA and MEHA/BSF-Eumel scaffolds were stable without showing mass loss over time for up to 21 days (Figure 6c,d). In particular, freeze-dried scaffolds with and without BSF-Eumel, upon contact with the rehydration water solution, were able to swell up to a value of 4000% during the first 30 min, reaching equilibrium as early as 1 h. There was no large difference between the kinetics of the two different kinds of 3D printed samples while considering the swelling profile over time, meaning that they reached their equilibrium at approximately the same time. However, MEHA/BSF-Eumel scaffolds demonstrated a decreased swelling ratio (*p* < 0.01) compared to neat materials (Figure 6c). This effect could be ascribed to the presence of BSF-Eumel sub-micrometer colloidal particles, which may restrict the chain mobility of MEHA. Hence, the highest swelling ratio found for the MEHA was in agreement with previous works [24,27]. The overall results demonstrated that the MEHA-based scaffolds swelled in the physiological solution maintaining their 3D lattice-like structure without collapsing over time, suggesting their suitability for tissue engineering applications. This aspect is of paramount importance, considering that scaffolds have to withstand external forces, before degrading and stimulating new tissue ingrowth.

Regarding the release profile of BSF-Eumel, it was continuously released from the 3D printed scaffolds during the tested period with 80% of BSF-Eumel being released after 3 days (Figure 6e). Another 15% of BSF-Eumel entrapped in the 3D printed sponge-like hydrogels was released between 3 and 15 days.

**Figure 7:**
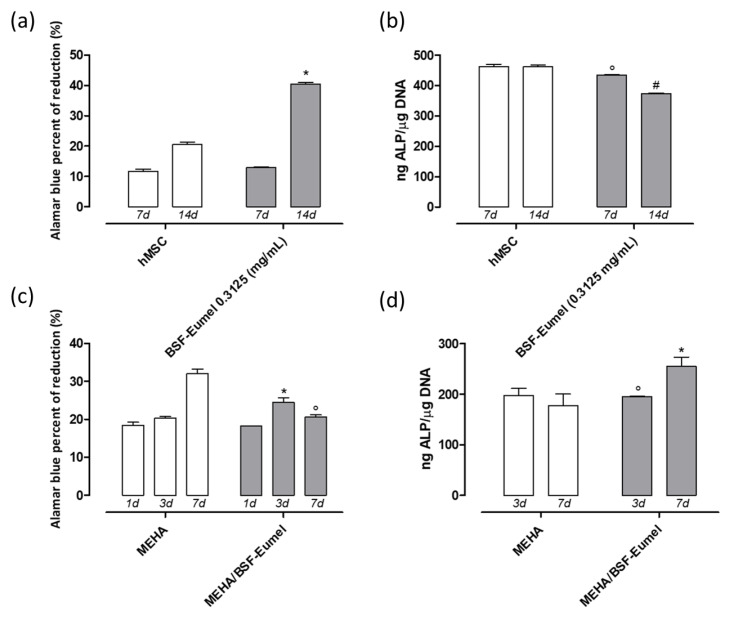
Cell proliferation and differentiation of hMSCs in contact with 0.3125 mg/mL of BSF-Eumel solution at 7 and 14 days of cell culture (**a**,**b**). Cell proliferation and differentiation of hMSCs seeded on MEHA- and MEHA/BSF-Eumel-based scaffolds after 1, 3 and 7 days of cell culture (**c**,**d**). * *p* < 0.05; ° *p* < 0.001; # *p* < 0.0001 *vs* control group.

Biological investigations highlighted a dual effect of BSF-Eumel sub-micrometer colloidal particles when incubated as PBS solution or incorporated into the 3D scaffolds (Figure 7). In particular, BSF-Eumel solution (0.3125 mg/mL) induced an increase in hMSCs viability at 14 days (Figure 7a) with a higher value of Alamar blue reduction than the control group (*p* < 0.05), whereas no significant activation of ALP was detected at the same time point (Figure 7b). When BSF-Eumel was embedded into the MEHA polymer matrix, higher values of cell viability were observed at 3 days (Figure 7c); moreover, MEHA/BSF-Eumel scaffolds were proven to promote ALP expression at 7 days of cell culture (*p* < 0.05) (Figure 7d). Overall, these data denote the high biocompatibility of BSF-Eumel and highlight the key role played by the pigment in reinforcing the osteogenic properties of the MEHA scaffold.

## 4. Conclusions

The method described in a recent patent [16] allowed the synthesis of significant amounts of pure eumelanins from black soldier fly cuticles, as evidenced by UV-vis and EPR spectra. The produced BSF-Eumel was characterized by a non-homogeneous sub-micrometer colloidal particle distribution and a good solubility in water and aqueous buffers. Furthermore, its high oxidation level may indicate the metal chelating attitude of the pigment. From a biological point of view, BSF-Eumel solutions were found to be biocompatible over a wide range of concentrations (0.05–2.5 mg/mL). At 0.3125 mg/mL, it was able to induce hMSCs’ proliferation even though it did not improve the ALP activity. As a proof of concept, MEHA/BSF-Eumel scaffolds were produced by 3D printing. Even if BSF-Eumel did not dramatically affect the physico-chemical and mechanical features of 3D printed scaffolds, it influenced the physiological response of hMSCs towards bone differentiation through the higher expression of the early osteogenic marker. Indeed, extruded MEHA/BSF-Eumel inks in lattice-like structures were shown to serve as biocompatible, self-supporting scaffolds for bone tissue regeneration, as evidenced by increased ALP activity.

These results pave the way for a new scenario in the field of eumelanin-based bio-interfaces prospecting the opportunity to exploit natural pigments for the fabrication of biomaterials. This perspective can capitalize on eumelanin’s ability to control biological responses, bypassing limits connected with the biomimetic synthesis of eumelanin and at the same time providing gains in terms of sustainability with respect to both farming and the circular economy from nature to regenerative medicine.

## Figures and Tables

**Table 1:** Elemental analysis of BSF-Eumel and 5,6-dihydroxyindole.

Samples	BSF-Eumel	5,6-Dihydroxyindole
**Carbon (C, % w)**	52.6	64.4
**Hydrogen (H, % w)**	5.8	4.7
**Nitrogen (N, % w)**	10.1	9.4
**Others (O, % w)**	31.5	21.5

## Data Availability

All the data have been reported in the paper.
